# Profile of Parent-Child Well-Being in Immigrant Families

**DOI:** 10.1093/geroni/igab046.768

**Published:** 2021-12-17

**Authors:** Mengting Li, Qun Le, XinQi Dong

**Affiliations:** 1 Rutgers, The State University of New Jersey, New Brunswick, New Jersey, United States; 2 Rutgers University, New Brunswick, New Jersey, United States; 3 Rutgers University, Rutgers Institute for Health, New Jersey, United States

## Abstract

Earlier caregiving research focused on psychological well-being of either caregivers or care recipients, while less is known about the caregiving pattern with optimal outcome for both caregivers and care recipients. Data were from the PINE and PIETY studies, with 804 parent-child dyads. Depressive symptoms were measured by PHQ-9 with a cutoff of 5 distinguishing happy or depressed. Parent-child dyads were divided into four groups: happy-parent-happy-child (HPHC, n=572, 71.1%), depressed-parent-happy-child (DPHC, n=139, 17.3%), happy-parent-depressed-child (HPDC, n=65, 8.1%), and depressed-parent-depressed-child (DPDC, n=28, 3.5%). Multinomial logistic regression was used to compare the sociodemographic differences among the groups. Compared to the HPHC group, the DPHC group had older parents, more mother-child dyads and lower-income children, the HPDC group had more female children. However, there was no significant difference between the HPHC and the DPDC group. Future research could explore the predictors of parent-child well-being to inform intervention strategies.

